# Effect of discharge planning rooted in the integrated Care for Older People Concept on the maintenance of intrinsic capacity in older adults

**DOI:** 10.3389/fpubh.2026.1838558

**Published:** 2026-10-16

**Authors:** Danni Zheng, Ren Xu, Shuhong Cai, Danfeng Pang, Xuemin Zhang, Yunxuan Zhu, Min Zhao

**Affiliations:** Department of Geriatrics, The First Affiliated Hospital of Ningbo University, Ningbo, Zhejiang, China

**Keywords:** older adults, comprehensive healthcare, geriatrics, patient discharge, randomised controlled trial

## Abstract

**Objective:**

To assess how integrated care for older people (ICOPE)-based discharge planning (DP) affects older patients’ intrinsic capacity (IC).

**Methods:**

Between December 2023 and June 2024, 112 geriatric inpatients were randomly assigned to control (routine care) or Experimental (routine care + ICOPE discharge services) groups. Intrinsic capacity domains were evaluated using the Short Physical Performance Battery, Saint Louis University Mental Status, Geriatric Depression Scale-Short Form and Mini Nutritional Assessment-Short Form tools and self-report at discharge and 3 months post-discharge. Analysis of covariance and binary logistic regression were used, with baseline scores, living status and gender as covariates. The primary analysis was supplemented by a sensitivity analysis using continuous domain scores as separate outcomes to verify the robustness of the composite score findings.

**Results:**

108 patients completed follow-up. Intrinsic capacity decline was lower in the experimental group (1.2 ± 0.2) than in the control group (3.1 ± 0.3; mean difference: –1.9, 95% CI: −2.5 to −1.3, *p* < 0.001). Motor, cognitive, psychological and nutritional domains improved more in the Experimental group (all *p* < 0.001); sensory function showed no difference (*p* = 0.267).

**Conclusion:**

The ICOPE-based DP approach slows IC decline and enhances multiple functional domains, offering a valuable reference for transitional care for older adults.

## 1 Introduction

China is currently the country with the largest older population and the fastest growth rate worldwide, with the ageing crisis escalating. Following the World Health Organization’s (WHO) introduction of the novel concept of healthy ageing, the intrinsic capacity (IC) of older adults has become a central focus of clinical research and practice. In 2015, WHO first defined IC as the integration of all physical and mental capabilities an individual can mobilise at any given time (1). In 2017, WHO proposed a five-dimensional model of IC, including mobility, vitality, cognition, sensory function and psychosocial function (2). This model fully captures changes in physical function and mental capacity within the healthy ageing framework and serves as a tool for rapid screening and assessment of functional status in older adults. Enhancing and maintaining IC in older adults is recognised as a key intervention to advance healthy ageing.

Contemporary research indicates that the incidence of IC decline among older patients ranges from 19.23 to 98.00%. This broad range not only reflects the high prevalence of IC impairment in the older population but also its variable severity, underscoring the urgency of early identification and intervention (3–6). The concept of integrated care for older people (ICOPE), originating in the United States, arose from the pressing societal demand to reform healthcare delivery models amid intertwined challenges: population ageing, shifts in disease patterns, growing complexity of older adults’ health and social needs and inadequate service provision by healthcare institutions. Its core components comprise patient empowerment, multidisciplinary team–based decision support, clinical information systems and coordination of nursing workflows (7). To translate the notion of healthy ageing from theory into practice, WHO released the *Guidelines on Integrated Care for Older People* (2); the guidelines and their accompanying handbooks centre on the management of IC decline in older adults and corresponding care pathways.

Furthermore, to enhance the utilisation of medical resources, the average length of hospital stay in healthcare facilities has been decreasing steadily over the years (8). Consequently, most older patients with chronic conditions must transition back to community and home settings for ongoing rehabilitation and care. Should these patients lack sufficient understanding of their condition or adequate home-based care preparation at discharge, they face a heightened risk of unplanned readmission due to suboptimal post-discharge care (9), which, in turn, exacerbates IC decline.

Discharge planning (DP), also known as the discharge preparation service programme, is a patient-centred theory. Upon patient admission, an assessment is conducted immediately to identify those with post-discharge care needs or at risk of delayed discharge. A multidisciplinary DP team collaboratively formulates a tailored post-discharge care plan for patients and their families or facilitates transfer to appropriate lower-level medical institutions or other geriatric care facilities. This enables patients and their families to leave the hospital with peace of mind while guaranteeing continuous and comprehensive medical and nursing care (10).

Based on the ICOPE concept, this study utilises DP as a medical care model. Upon patient admission, assessments were conducted immediately on the five dimensions of IC in older adults. Individualised interventions were developed and implemented based on the assessment results of each dimension for individual patients. Discharge planning is a collaborative initiative involving healthcare professionals, patients, their families and primary caregivers, ensuring that patients receive comprehensive and continuous care both during hospitalisation and post-discharge. Through a closed-loop management model, the continuity of geriatric care is achieved, establishing a hospital–community–family tripartite collaborative model. This study explores the observed impact and delaying effect of DP on IC decline in older patients, providing references for clinical nursing practice.

## 2 Participants and methods

### 2.1 Study design

The study was conducted in strict compliance with the Declaration of Helsinki and relevant national ethical guidelines for medical research and has been approved by the Ethics Committee of the First Affiliated Hospital of Ningbo University (Approval No.: L2023Y No. 193A). This study has been registered in the ISRCTN registry (Registration Number: ISRCTN31570632).

Based on pilot study findings (the IC decline score in the control group was 3.2 ± 1.7, and that in the Experimental group was 1.6 ± 1.5; the mean difference between the two groups was 1.6, and the combined standard deviation was 1.6), a standard deviation (*σ*) of 1.8 for the IC decline score, a mean difference (*δ*) of 1.5 between the two groups, *α* = 0.05 (two-tailed) and *β* = 0.20 were assumed. The sample size was calculated using the following formula for comparing means of two independent samples: *n* = 2(*Zα*/2 + *Zβ*)^2^*σ*^2^/*δ*^2^. This calculation yielded a required sample size of 48 cases per group. Accounting for a 10% loss to follow-up rate, the final sample size was set at 56 cases per group, with a total of 112 cases.

### 2.2 Study participants

A total of 112 older inpatients admitted to the geriatrics department of our hospital between December 2023 and June 2024 were enrolled. Randomisation was performed using a random number table: 112 random numbers were generated by a researcher who was not involved in recruitment and intervention implementation using SAS 9.4 software (SAS Institute Inc., Cary, NC, USA), sorted in ascending order and assigned sequential numbers from 1 to 112. Participants with numbers 1–56 were allocated to the Experimental group, and those with numbers 57–112 to the control group. Random assignment cards were printed according to the sequential random number and grouping information, then sealed in opaque envelopes. Envelopes were prepared and administered by another researcher who was not involved in the trial. When each participant was included, the researcher responsible for enrolment opened the envelopes in order of envelope number and implemented the corresponding intervention according to the assigned group. Outcome assessors and statistical analysts were blinded to group allocation status. Due to the nature of the intervention, blinding of participants and intervention providers was not possible, but potential bias was minimised by using a standardised intervention protocol and independent outcome assessments. Participants were randomly assigned to the control group (*n* = 56) and the Experimental group (*n* = 56) in a 1:1 ratio. All participants provided written informed consent.

*The inclusion criteria were as follows*: (1) age ≥65 years, (2) hospitalised in the department of geriatrics, (3) adequate communication abilities to cooperate with surveys and related assessments, (4) provision of written informed consent and voluntary participation and (5) expected survival time >6 months.

*The exclusion criteria included the following*: (1) end-stage disease with an expected survival time ≤6 months, (2) acute and critical conditions and (3) severe dementia or severe disability precluding completion of the assessments.

*The termination criteria were as follows*: (1) occurrence of serious adverse events in participants during the study; (2) significant deterioration of the participant’s condition requiring modification of the treatment regimen, affecting the implementation of the intervention; (3) voluntary withdrawal from study participation; and (4) loss to follow-up (failure to complete two consecutive follow-ups with unsuccessful attempts to contact the participant). Following termination of participation, participants were managed and followed up in accordance with clinical and ethical guidelines, and data from all terminated cases were included in the intention-to-treat (ITT) analysis.

## 3 Study methods

### 3.1 Control group

Upon admission, electronic health records were developed based on the hospital’s initial nursing assessment form for inpatients. During hospitalisation, routine disease-specific nursing interventions and health education were administered. Discharge was arranged per medical orders. Prior to discharge, disease-related nursing education was provided, including dietary, medication and exercise guidance. Patients were informed of follow-up requirements. 1 week post-discharge, primary nurses conducted telephone follow-ups to assess rehabilitation progress, adherence to medical advice regarding diet management, medication administration and tube care, and to reinforce recovery-promoting behaviours and promptly correct behaviours adverse to recovery. Through communication, potential safety risks, such as falls and pressure ulcers during self-care, were identified, and timely extended nursing interventions were provided. Other care-related inquiries were also addressed. Patients were advised to return to the Geriatric Specialty Nursing Clinic for follow-up assessment 3 months after discharge.

### 3.2 Experimental group

In addition to the routine interventions administered to the control group, the DP was implemented following the five-dimensional systematic pathway outlined in the ICOPE guidelines. Intervention records were fully traceable throughout the process, with implementation and follow-up conducted collaboratively by an interdisciplinary team.

1 *Establishment of a DP team*: The team was composed of two geriatricians, four nurses (including two geriatric specialist nurses, one care trainer and one head nurse), one dietitian, one rehabilitation therapist and one pharmacist. The head nurse served as the liaison for DP, tasked with coordinating all aspects of the service to ensure its efficient, effective and continuous delivery (11). All team members possessed over 5 years of clinical experience. A 2-month pre-service training programme on DP was implemented to equip members with essential skills, and all successfully completed the corresponding assessments. Following team formation, members were prohibited from withdrawing without just cause to ensure the work progressed smoothly.
2 *Development and implementation of plans*: Standardised DP and intervention protocols were formulated based on the comprehensive assessment results by specialist nurses and the *Guidelines for Community Interventions to Manage Decreased Intrinsic Capacity in Older Adults* issued by WHO:

- *Mobility*: Based on the results of the Short Physical Performance Battery (SPPB) assessment, patients with a score ≤8 (12) were provided with a standardised exercise plan developed by the team, comprising resistance training three times weekly, 30 min per session. Lower limb training involved elastic bandages of the same specification, whereas upper limb training consisted of dumbbell-like lifts using one 550-ml mineral water bottle per hand. A standardised resistance training video was created as a reference template for discharged patients.
- *Vitality (nutrition)*: For patients with a score ≤11 on the Mini Nutritional Assessment-Short Form (MNA-SF) (13), dietitians performed weekly follow-ups to deliver individualised dietary counselling and a nutrient composition conversion table to discharged patients.
- *Sensory function*: Enhanced assessment and support were provided to patients who reported visual or auditory impairment impacting daily activities. For patients requiring ophthalmic drops, standardised medication counselling was delivered to ensure proper administration, and a standardised eye exercise video was developed for uniform guidance. Patients with hearing impairment were promptly advised to utilise hearing aids.
- *Cognitive function*: For patients with cognitive impairment, specialist nurses designed multimodal cognitive stimulation therapy, comprising weekly group therapy during hospitalisation and twice-daily home-based cognitive training sessions (10 min per session). In-hospital weekly group therapy consisted of groups of 5–8 patients to ensure interaction (45–60 min per session). Activities included cognitive stimulation (number solitaire, word association, picture memory matching, simple puzzles) targeting attention, memory and language; reality orientation training (collective newspaper reading, discussions on date/weather/current events) to maintain time–space awareness; and experience-based interactive learning (recalling occupational skills, sharing family traditions) to activate long-term memory. Medical staff adjusted activity difficulty dynamically (e.g., more complex tasks for mild impairment, simplified rules for moderate impairment). For the twice-daily home-based cognitive training (10 min/session), pre-discharge, specialist nurses provided one-on-one training to patients and primary caregivers (with standardised manuals and demonstrations). Content combined mandatory standardised modules (memory training [memorising 3–5 items, recalling meals/activities], attention training [timed number cancellation, illustrated mazes], logical thinking training [adaptive arithmetic, household item classification]) and personalised modules (language training for language impairment, building block assembly/item placement memory for visual–spatial decline). Training was scheduled at fixed times (e.g., 30 min post-meals) to avoid fatigue. Caregivers supervised, recorded the progress and provided feedback to the medical team via WeChat; medical staff adjusted content through weekly follow-ups.
- *Psychosocial function*: For patients with a score ≥8 on the Geriatric Depression Scale-Short Form (GDS-15, 14), music therapy, reminiscence therapy, narrative nursing and emotional nursing were implemented. Furthermore, patients’ medication regimens were documented, a medication list was developed and patients were educated on medication administration, therapeutic effects and adverse reactions.

Prior to discharge, the primary caregiver was identified. The discharge plan manual was provided to the patient, their family members and the primary caregiver with detailed instructions, and their mastery of the content was reassessed at discharge. A WeChat group for DP was established, where professionals addressed inquiries and dynamically monitored adherence to interventions. Patients’ post-discharge destinations were confirmed, and patient data along with relevant post-discharge care plans were provided to receiving institutions to establish collaborative links. The Discharge Preparation Plan Checklist (see Appendix I) was finalised within 48 h prior to discharge.

3 *Post-discharge follow-up*: 1 week post-discharge, primary nurses conducted telephone follow-ups and utilised the WeChat group for DP to monitor patients’ status in real time. Subsequent follow-ups were performed biweekly for a total of 3 months, with content aligned with the discharge plan manual, including assessments of medication adherence, mastery of fall prevention knowledge, proficiency in pressure ulcer prevention, primary caregiver competence and adherence to rehabilitation exercises. At 3 months post-discharge, patients were instructed to return to the Geriatric Specialty Nursing Clinic for reassessment of IC decline.

### 3.3 Outcome measures

1 *Baseline data*: Compiled by the research team based on a review of relevant literature (9–11), including age, gender, educational level, living status, marital status, monthly family income, number of medications used, alcohol consumption and smoking status, past medical history, medication adherence, mastery of fall prevention knowledge, proficiency in pressure ulcer prevention skills, care capacity of primary caregivers and adherence to rehabilitation exercises (see Appendix II). These data were collected via questionnaires and electronic medical record reviews by the research team following patient admission.

Outcome measures included the total IC score and raw scores of its five dimensions (mobility, vitality, cognition, sensory function and psychosocial function), assessed at admission (baseline) and 3 months post-discharge. Follow-up data were captured by primary nurses via face-to-face assessments, telephone follow-ups and real-time documentation through the discharge WeChat group, ensuring data completeness and traceability.

2 *Primary outcome measures*:

The primary outcomes were the raw scores of four continuous IC domains: mobility, cognition, psychosocial function, and vitality. These were assessed using validated instruments at baseline (within 48 h of admission) and 3 months post-discharge. Mobility was assessed using the SPPB (12), a performance-based test with scores ranging from 0 to 12, where higher scores indicate better function (Cronbach’s *α* > 0.80). Nutritional status (vitality) was assessed with the MNA-SF (13), a 14-point screening tool where higher scores reflect better nutritional status (Cronbach’s *α*: 0.70–0.90). Psychosocial function was evaluated using the GDS-15 (14), a self-reported measure of depressive symptoms (score range 0–15, higher scores indicate more severe depression, Cronbach’s *α*: 0.75–0.88). Cognition was measured with the SLUMS examination (15), a 30-point scale covering orientation, memory, attention, and executive function, with good internal consistency (Cronbach’s *α*: 0.84–0.88).

3 *Secondary outcome measures*:

- *Sensory function*: A self-report assessment was employed, primarily evaluating whether visual or auditory impairment impacted daily activities. A score of 1 was assigned for the presence of such impairment and 0 for the absence of such impairment. This assessment approach has been widely adopted in the WHO ICOPE guidelines.
- *Exploratory composite IC score*: Based on the evaluation framework of the National Geriatric Medical Center, with reference to WHO’s definition of the five IC dimensions, and integration of relevant domestic and international medical guidelines, consensuses and high-quality literature (16, 17), a five-dimensional composite scoring system was established as an exploratory tool. A score of 1 was assigned for the decline in each dimension, resulting in a total score ranging from 0 to 5. A score of 0 indicates no IC decline, whereas higher scores indicate more significant IC decline (18). The composite measure was validated in a pilot study with a limited sample of 30 older inpatients. An exploratory factor analysis (using principal component analysis with varimax rotation) was performed, yielding a Kaiser–Meyer–Olkin measure of 0.72 and a significant Bartlett’s test of sphericity (*p* < 0.001). The tool demonstrated acceptable item discrimination (item-total correlations: 0.45–0.78) and internal consistency (Cronbach’s *α* = 0.78). We explicitly present this composite score as an exploratory measure due to the pilot study’s sample size being insufficient for confirmatory factor analysis. Its primary role in this study is to complement the primary continuous domain analyses by providing a summative indicator of overall IC decline.

Follow-up data were captured by primary nurses via face-to-face assessments, telephone follow-ups and real-time documentation through the discharge WeChat group, ensuring data completeness and traceability.

### 3.4 Statistical methods

Statistical analyses were conducted using SPSS 26.0 software (IBM Corp., Armonk, NY, USA). All continuous variables were first tested for normality using the Kolmogorov–Smirnov test (sample size ≥50). Measurement data that followed a normal distribution were presented as mean ± standard deviation (x ± s), whereas non-normally distributed data were expressed as median (interquartile range). Categorical data were reported as percentages (%). Continuous variables were compared between groups using analysis of covariance (ANCOVA), with baseline scores, living status, and gender entered as covariates to adjust for potential baseline imbalances. Adjusted means and standard errors are reported for post-intervention outcomes. For non-normally distributed continuous variables, the robustness of the ANCOVA results was confirmed by examining residual plots; sensory function (categorical) was analyzed using binary logistic regression with the same covariates. Intra-group pre-post comparisons were performed using the Wilcoxon signed-rank test. To control for Type I error inflation due to multiple comparisons, Bonferroni correction was applied separately for inter-group comparisons (6 tests; *α*’ = 0.0083) and intra-group comparisons (6 tests per group; *α*’ = 0.0083). A two-tailed *p*-value <0.05 was considered statistically significant before correction. To assess the robustness of our findings based on the exploratory composite score, we conducted a sensitivity analysis by re-running the primary between-group comparisons using the continuous domain scores as separate outcomes, which confirmed the direction and statistical significance of the results.

## 4 Results

### 4.1 Comparison of baseline characteristics between the two groups

The research process is shown in Figure 1. During the 3-month follow-up period, four participants (3.6%) discontinued, two in the Experimental group (one due to disease progression requiring treatment modification at week 3 and one who withdrew consent at week 2) and two in the control group (one lost to follow-up after week 5 despite three contact attempts and one who experienced disease progression at week 4). No participants were excluded from ITT analysis. The final ITT analysis included all 112 randomised participants, with missing 3-month outcome data imputed using the last observation carried forward method. A complete-case analysis was performed on the 108 participants who completed the 3-month assessment (54 per group). Baseline characteristics of completers versus non-completers showed no significant differences (all *p* > 0.05).

**Figure 1:**
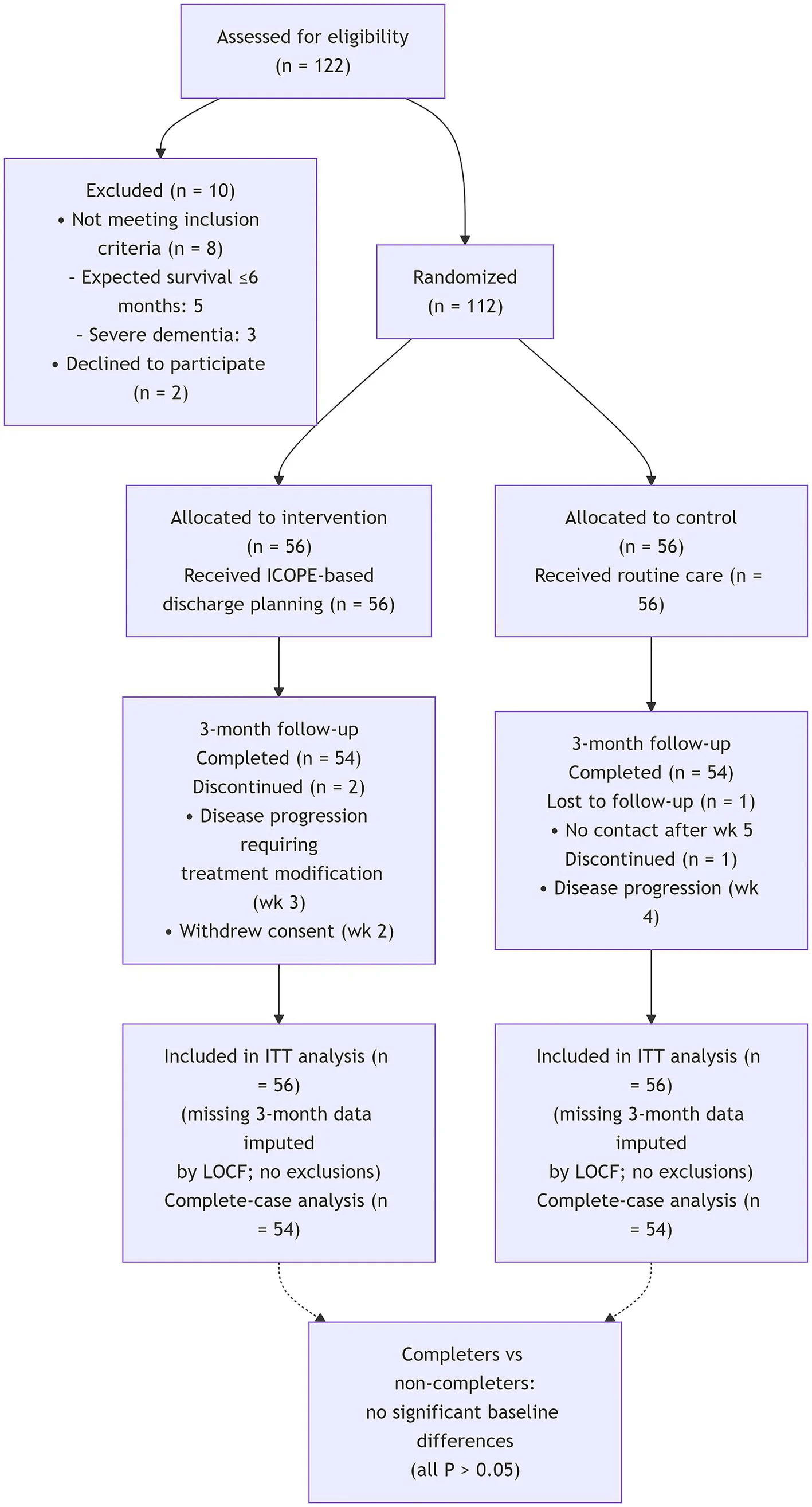
Flowchart of subject screening, randomization, and follow-up.

No statistically significant differences were observed in baseline characteristics between the two groups, including age, body mass index, gender, educational level, living status, monthly family income, number of medications used, marital status, alcohol consumption and smoking status, prevalence of hypertension, medication adherence, mastery of fall prevention knowledge, proficiency in pressure ulcer prevention skills, care capacity of primary caregivers and adherence to rehabilitation exercises (all *p* > 0.05), indicating good comparability (Table 1).

**Table 1:** Comparison of baseline data between the two groups.

Variable	Category	Experimental group (*n* = 56)	Control group (*n* = 56)	*t*/*u*/χ^2^	*p*
Age		75.52 ± 5.53	76.14 ± 5.40	−0.605	0.546
Body Mass Index (BMI)		23 (20–25)	23 (20–24)	1575.5	0.967
Gender	Male	37 (66.1%)	29 (51.8%)	1.808	0.179
	Female	19 (33.9%)	27 (48.2%)		
Educational level	Illiterate	5 (8.9%)	6 (10.7%)		0.583
	Primary school or below	10 (17.9%)	8 (14.3%)		
	Middle school	18 (32.1%)	14 (25.0%)		
	High school	14 (25.0%)	12 (21.4%)		
	College or above	9 (16.1%)	16 (28.6%)		
Living status	Living alone	14 (25.0%)	24 (42.9%)	3.226	0.073
	Not living alone	42 (75.0%)	32 (57.1%)		
Monthly family income	3,000 ~ 5,000	28 (50.0%)	23 (41.1%)		0.518
	>5,000	15 (26.8%)	20 (35.7%)		
	<3,000	13 (23.2%)	13 (23.2%)		
Number of medications used	<5	25 (44.6%)	27 (48.2%)	0.036	0.85
	≥5	31 (55.4%)	29 (51.8%)		
Marital status	Married	44 (78.6%)	42 (75.0%)		0.524^*^
	Divorced	5 (8.9%)	3 (5.4%)		
	Widowed	7 (12.5%)	11 (19.6%)		
Alcohol consumption	No	34 (60.7%)	33 (58.9%)	0	1
	Yes	22 (39.3%)	23 (41.1%)		
Smoking status	No	38 (67.9%)	40 (71.4%)	0.042	0.837
	Yes	18 (32.1%)	16 (28.6%)		
Hypertension status	No	26 (46.4%)	21 (37.5%)	0.587	0.444
	Yes	30 (53.6%)	35 (62.5%)		
Medication adherence	Good	39(69.6%)	37(66.1%)	χ^2^ = 0.102	0.749
	Fair	14(25.0%)	15(26.8%)		
	Poor	3 (5.4%)	4 (7.1%)		
Mastery of fall prevention knowledge	Good	35 (62.5%)	33 (58.9%)	χ^2^ = 0.128	0.72
	Fair	17 (30.4%)	18 (32.1%)		
	Poor	4 (7.1%)	5 (8.9%)		
Proficiency in pressure ulcer prevention skills	Good	33 (58.9%)	31 (55.4%)	χ^2^ = 0.105	0.746
	Fair	19 (33.9%)	20 (35.7%)		
	Poor	4 (7.1%)	5 (8.9%)		
Care capacity of primary caregivers	Good	36 (64.3%)	34 (60.7%)	χ^2^ = 0.145	0.703
	Fair	17 (30.4%)	18 (32.1%)		
	Poor	3 (5.4%)	4 (7.1%)		
Adherence to rehabilitation exercises	Good	34 (60.7%)	32 (57.1%)	χ^2^ = 0.136	0.713
	Fair	18 (32.1%)	19 (33.9%)		
	Poor	4 (7.1%)	5 (8.9%)		

For marital status, Fisher’s exact test was used due to expected cell frequencies <5, indicated by *.

### 4.2 Inter-group comparison of measures before and after intervention between the two groups

At baseline, no significant differences were observed between the experimental and control groups in SPPB, SLUMS, GDS-15, MNA-SF scores, or sensory function (all *p* > 0.05). Following intervention, after adjusting for baseline imbalances in living status and gender, the experimental group demonstrated significantly better outcomes in all primary continuous measures compared with the control group: SPPB (adjusted mean difference [aMD] = 1.8, 95% CI: 1.3–2.3, *p* < 0.001), SLUMS (aMD = 3.1, 95% CI: 2.3–3.9, *p* < 0.001), GDS-15 (aMD = −1.6, 95% CI: −2.1 to −1.1, *p* < 0.001), and MNA-SF (aMD = 1.4, 95% CI: 1.0–1.8, *p* < 0.001; Table 2). No significant difference in sensory function was observed between the two adjusted groups (*p* = 0.066).

**Table 2:** Intergroup comparison of indicators between the Experimental Group (*n* = 56) and Control Group (*n* = 56).

Outcome indicator	Group	Experimental	Control	Test statistic (*t*/*u*/χ^2^/F)	*p*-value
Short Physical Performance Battery (SPPB) [M (IQR)]	Before intervention	9 (8–9)	8.50 (8–9)	1518.500	0.761
	After intervention	10 (9–11)	8 (8–9)	89.619	<0.0001
The Saint Louis University Mental Status Examination (SLUMS) (x ± s)	Before intervention	22.98 ± 2.32	22.38 ± 1.77	0.512	0.609
	After intervention	25.54 ± 2.64	22.62 ± 1.95	175.136	<0.0001
Geriatric Depression Scale-15 (GDS-15) [M (IQR)]	Before intervention	5.50 (4–7)	6 (4.75–7)	1575.000	0.969
	After intervention	4 (3–5.25)	5.50 (4–7)	65.687	<0.0001
Mini Nutritional Assessment-Short Form (MNA-SF) [M (IQR)]	Before intervention	11 (11–12)	12 (11–12)	1364.000	0.216
	After intervention	13 (12–14)	12 (11–12)	112.266	<0.0001
Sensory Function (Decline, *n*, %)	Before intervention	47 (83.9%)	46 (82.1%)	0.000	1.000
	After intervention	45 (80.4%)	46 (82.1%)	3.439	0.066
Total Intrinsic Capacity Score[M (IQR)]	Before intervention	3 (2–3)	2 (2–3)	1575.500	0.967
	After intervention	1 (1–2)	3 (2–3)	63.503	<0.0001

(1). Measurement data following normal distribution are expressed as mean ± standard deviation (x ± s), and compared using the *t*-test; non-normally distributed data are expressed as median (interquartile range) [M (IQR)], and compared using the Mann–Whitney *U* test. (2). Categorical data (sensory function decline) are expressed as number (percentage) [*n*, (%)], and compared using the χ^2^ test. (3). Bonferroni correction was applied for 6 inter-group comparisons; statistical significance was set at *p* < 0.0083. All reported *p*-values are Bonferroni-corrected”.

For the exploratory composite IC score, the experimental group also showed a significantly lower decline score compared with the control group (1.2 ± 0.2 vs. 3.1 ± 0.3, mean difference: −1.9, 95% CI: −2.5 to −1.3, *p* < 0.001). The sensitivity analysis, which used the continuous domain scores as separate outcomes in the primary ANCOVA models, confirmed the robustness of these findings, showing consistent direction and significance (all *p* < 0.001).

### 4.3 Intra-group comparison of measures before and after intervention in each group

In the experimental group, following 3 months of intervention, SPPB, SLUMS and MNA-SF scores were significantly increased, whereas GDS-15 score was significantly reduced (all adjusted *p* < 0.001). No significant difference in sensory function was detected before and after intervention (adjusted *p* = 0.424). In the control group, no significant changes were observed in SPPB, GDS-15, MNA-SF, or sensory function before and after intervention (all adjusted *p* > 0.05). Only the SLUMS score exhibited a modest change, which was not statistically significant following Bonferroni correction (adjusted *p* = 0.015, corrected *p* > 0.05). Notably, the median SPPB score in the control group dropped from 8.5 to 8, indicating a natural decline in patients’ mobility under routine care (Table 3). For the exploratory composite IC score, the experimental group showed a significant reduction (*p* < 0.001), while the control group showed no significant change (*p* = 0.360).

**Table 3:** Intragroup comparison of indicators in each group.

Group	Variable	Before intervention	After Intervention	*z*	*p*
Control group	SPPB	8.50 (8–9)	8 (8–9)	42	0.151
	SLUMS	23 (21–24)	24.12 (23–25)	28	0.015^[note 1]^
	GDS15	6 (4.75–7)	5.50 (4–7)	48	0.251
	MNASF	12 (11–12)	12 (11–12)	67.5	0.978
	Total intrinsic capacity score	2 (2–3)	3 (2–3)	39	0.360
	Sensory function (decline)	46 (82.1%)	46 (82.1%)	χ^2^ = 0.000	1.000
Experimental group	SPPB	9 (8–9)	10 (9–11)	0	<0.001
	SLUMS	23 (21–25)	25.50 (24–28)	0	<0.001
	GDS15	5.50 (4–7)	4 (3–5.25)	0	<0.001
	MNASF	11 (11–12)	13 (12–14)	0	<0.001
	Total intrinsic capacity score	3 (2–3)	1 (1–2)	24	<0.001
	Sensory function (decline)	47 (83.9%)	51 (91.1%)	χ^2^ = 0.636	0.424

(1). Intra-group comparisons used Wilcoxon signed-rank test for non-normally distributed data, with values presented as median (IQR). (2). Bonferroni correction was applied for 6 intra-group comparisons per group; statistical significance was set at *p* < 0.0083. Only the Experimental group showed significant improvements in all domains (*p*_corr < 0.001). The modest change in SLUMS in the control group was not significant after correction (*p*_corr = 0.09).

### 4.4 Adverse events

No intervention-related or serious adverse events were reported in either group during the study period.

## 5 Discussion

The IC of older adults is oriented towards achieving the goal of healthy ageing. Its five dimensions—cognition, psychosocial function, sensory function, vitality and mobility—are intertwined and mutually influential, functioning synergistically. The developmental trajectory of IC is dynamically reversible (19). These five dimensions transform IC from a broad concept into quantifiable, monitorable indicators (20), enabling the screening and identification of deficient IC dimensions in older adults (21). Early identification of decreased IC and timely implementation of targeted interventions to maintain or even reverse IC are of great significance for improving the quality of life of older adults and promoting healthy ageing.

In clinical practice, exploring interventions to preserve IC involves tracking and monitoring this construct at both individual and population levels. Guided by a novel healthcare model centred on service integration and multidisciplinary collaboration, efforts prioritise person-centred care plans and individualised nursing interventions to reverse, mitigate or prevent IC loss (22). Integrating the ICOPE concept into clinical practice—with DP as the entry point—represents a novel initiative in our exploration of IC preservation in older adults. The World Health Organization defines integrated care as coordinated, proactive and person-centred multidisciplinary care delivered by two or more well-communicating and collaborative service providers, either within or across sectors (e.g., medical institutions, social care facilities and communities) (23). Discharge planning has been well established and widely implemented in countries such as the United States, the United Kingdom and Japan, as well as the Taiwan region of China (24–26). To address population ageing, control public medical expenditures and ensure the quality of health services, countries such as the United States and the United Kingdom have continuously emphasised concepts such as DP and continuity of care, as well as their importance for the effective utilisation of health resources (27). The DP model is a coordinated, centralised and multidisciplinary team–integrated process that requires collaboration among healthcare professionals, patients and their families to ensure that all patients receive continuous and comprehensive post-discharge care. Statistics indicate that only 15% of older adults reside in hospitals, whereas 85% receive care in community settings. Conversely, 98% of nurses are employed in hospitals, resulting in an extremely low proportion of nurses in communities, primary care settings and nursing homes, as well as a significant staffing shortage. This underscores the urgent need to refine DP to efficiently address referral needs and enhance the quality of continuous care. As a key component of effective continuous care, DP effectively connects in-hospital and out-of-hospital care, ensuring the sustained maintenance and assessment of all five IC dimensions.

Based on the ICOPE concept, this study validated the efficacy of DP in mitigating IC decline among older inpatients through multidisciplinary collaboration and sustained support. Our findings revealed balanced baseline characteristics between the two groups. At the 3-month follow-up, the Experimental group exhibited a significantly lower total IC decline score, alongside substantially greater improvements in mobility, cognitive function, psychological status and nutritional status compared with the control group. This aligns with WHO’s proposition regarding the dynamic reversibility of IC, underscoring the pivotal role of early targeted interventions in preserving functional status among older patients. While the composite score provides a useful summative indicator, the robust findings from the continuous domain scores, which are less susceptible to information loss and categorization bias, strengthen the validity of our conclusions.

A primary strength of DP lies in its systematicity and continuity. In contrast to conventional nursing care, the service integrates multidimensional assessments, implements individualised interventions and leverages multidisciplinary team collaboration to accurately identify patients’ functional deficits. This approach ensures the comprehensiveness and scientific rigour of interventions, thereby facilitating the optimal timing of discharge and delivering high-quality discharge guidance to patients. Currently, the timing of conventional discharge is solely determined by physicians’ medical orders. In contrast, the DP model adopts a joint decision-making process between physicians and nurses, incorporating not only physicians’ medical assessments but also considerations of continuous care, social and family support as well as psychological factors, making the process more rigorous. Conventional clinical discharge procedures may create a communication gap between healthcare providers and older patients, overlooking the individualised discharge needs of older adults. Due to constraints such as heavy workloads and limited time, healthcare professionals, especially nurses, are required to provide extensive guidance and interventions to patients in a short period before discharge, resulting in hasty and inadequate discharge education for older patients. Conversely, the planned implementation of DP involves assessing the five dimensions of IC immediately after admission, enabling the collection of comprehensive patient data. As key members of the DP team, nurses undergo a 2-month specialised training programme, which enhances their competence in discharge care guidance, health education (including content, methods and tools) and communication skills, thereby improving the quality of discharge education. Through continuous training and re-assessment of the five dimensions of IC during hospitalisation, the team gains a more in-depth understanding of patients’ conditions, making post-discharge extended care more targeted and personalised, with clear goals and strong operability. Additionally, the service confirms patients’ post-discharge destinations; provides the developed discharge preparation plans to patients, their families and primary caregivers; conducts regular follow-ups to monitor the implementation of interventions; and achieves closed-loop management and more efficient continuous care.

Second, DP achieves seamless connectivity between hospitals, communities and families through tools such as discharge manuals and WeChat group follow-ups, further enhancing intervention continuity. Conventional discharge education often lacks information on follow-up appointments for outpatient and community services, leaving patients vulnerable to anxiety post-discharge. A qualitative study exploring older adults’ transitional experiences noted that most caregivers lack knowledge of community services; their unfamiliarity with available resources undermines both continuity of care and patients’ rehabilitation outcomes. In contrast, DP provides caregivers with care-related knowledge during both hospitalisation and discharge guidance. Prior to discharge, patients are informed of community resources and the scope of social services, and assistance is provided in contacting receiving institutions, meeting the needs of patients and caregivers for care transition during hospitalisation and following discharge. For patients transferred to receiving institutions, patient data and post-discharge plans related to IC maintenance (including medication, diet, exercise and rehabilitation) are provided to ensure the continuity and sharing of information, thereby guaranteeing the continuity of nursing services. Furthermore, through the discharge preparation WeChat group, healthcare guidance remains uninterrupted, and patient and family questions are addressed promptly. A comprehensive post-discharge follow-up plan is implemented, including follow-up at the Geriatric Specialty Nursing Clinic, with specified follow-up timing, content and evaluation of the outcomes of IC maintenance. The decrease in SPPB scores in the control group of this study also confirms the natural decline in IC in older patients following discharge, making the Experimental group’s improvement effect particularly important.

We acknowledge the baseline imbalance regarding living status and gender between the two groups. Although these differences were not statistically significant (*p* > 0.05), they are clinically meaningful. To ensure the robustness of our findings, we included these variables as covariates in the ANCOVA model. The significant differences in IC improvement and its domains persisted even after this adjustment, suggesting that the observed benefits of the ICOPE-based DP are independent of these demographic factors. Furthermore, the higher proportion of patients living alone in the control group (who typically have less social support) might have actually made it more difficult for the control group to show improvement, thereby making our positive intervention results even more compelling.

This study is the first to integrate the ICOPE concept with DP, addressing a critical gap in domestic research. By combining multidisciplinary collaboration with individualised, sustained interventions, we developed a systematic, evidence-based and feasible intervention protocol. The integration of closed-loop management and hospital–community–family connectivity enhances the intervention’s sustainability and scalability.

Nevertheless, this study has certain limitations. First, the sample size was relatively small, and the follow-up period was only 3 months, limiting the evaluation of long-term effects. Second, sensory function was assessed using a single self-assessment question rather than objective tools such as visual acuity or hearing tests, which may have led to measurement bias, a major weakness in this methodology. Third, the IC composite score we used is an exploratory tool. While it demonstrated acceptable internal consistency in our pilot study, the sample size (*n* = 30) was insufficient for rigorous confirmatory factor analysis. We therefore placed primary emphasis on the validated continuous domain scores (SPPB, SLUMS, GDS-15, MNA-SF) as our main outcomes, and the composite score served only as a supplementary indicator. The consistent findings from both the primary continuous measures and the sensitivity analysis reinforce the robustness of our conclusions. Future research should employ well-validated composite IC assessment tools. Fourth, there was a difference in follow-up frequency between the intervention and control groups (the Experimental group had more frequent visits), which may have led to an ‘attention effect’ that amplified the intervention effect. Finally, the intervention programme of this study relied on abundant multidisciplinary resources (including rehabilitation therapists, nutritionists and pharmacists) and 2 months of team training, which may be difficult to promote in primary medical institutions with limited resources.

## Data Availability

The original contributions presented in the study are included in the article/supplementary material, further inquiries can be directed to the corresponding authors.
